# The Influence of *Fusarium* Mycotoxins on the Liver of Gilts and Their Suckling Piglets

**DOI:** 10.3390/ani11092534

**Published:** 2021-08-28

**Authors:** Tamara Dolenšek, Tanja Švara, Tanja Knific, Mitja Gombač, Boštjan Luzar, Breda Jakovac-Strajn

**Affiliations:** 1 Institute of Pathology, Wild Animals, Fish and Bees, Veterinary Faculty, University of Ljubljana, Gerbičeva ulica 60, 1000 Ljubljana, Slovenia; tanja.svara@vf.uni-lj.si (T.Š.); mitja.gombac@vf.uni-lj.si (M.G.); 2 Institute of Food Safety, Feed and Environment, Veterinary Faculty, University of Ljubljana, Gerbičeva ulica 60, 1000 Ljubljana, Slovenia; tanja.knific@vf.uni-lj.si (T.K.); breda.jakovac-strajn@vf.uni-lj.si (B.J.-S.); 3 Institute of Pathology, Faculty of Medicine, University of Ljubljana, Korytkova 2, 1000 Ljubljana, Slovenia; bostjan.luzar@mf.uni-lj.si

**Keywords:** *Fusarium* mycotoxins, pig, liver, histopathology, immunohistochemistry, apoptosis, proliferation index

## Abstract

**Simple Summary:**

Mycotoxins are toxic secondary metabolites of fungi that frequently contaminate animal feed and human food in different combinations; therefore, it is of great importance to determine the effects of mycotoxin co-contamination. Pigs are one of the most sensitive animal species to *Fusarium* mycotoxins, and the liver is an important site of mycotoxin metabolism. The objective of the present research was to determine histopathological changes, apoptosis, and proliferation in the liver of gilts fed with *Fusarium* mycotoxin-contaminated feed for a prolonged time at the end of their pregnancy and until weaning of their piglets. Additionally, the same parameters were evaluated in the liver of their piglets to determine whether *Fusarium* mycotoxins would affect the offspring. The results revealed increased hepatocellular necrosis and apoptosis as well as sinusoidal leukocytosis with inflammatory infiltrates of hepatic lobules in experimental gilts, but no significant changes were observed in the piglet livers, implying that the utilized concentrations and duration of exposure did not cause detrimental effects on them. Interestingly, the amount of interlobular connective tissue in the liver of experimental gilts was significantly decreased. The obtained results emphasized the need to evaluate *Fusarium* mycotoxin concentrations in feed because even at low concentrations, they can cause adverse effects, but there is less concern for severe detrimental effects on the offspring.

**Abstract:**

Mycotoxins are common fungal secondary metabolites in both animal feed and human food, representing widespread toxic contaminants that cause various adverse effects. Co-contamination with different mycotoxins is frequent; therefore, this study focused on feed contaminated with *Fusarium* mycotoxins, namely, deoxynivalenol (5.08 mg/kg), zearalenone (0.09 mg/kg), and fusaric acid (21.6 mg/kg). Their effects on the liver of gilts and their piglets were chosen as the research subject as pigs are one of the most sensitive animal species that are also physiologically very similar to humans. The gilts were fed the experimental diet for 54 ± 1 day, starting late in their pregnancy and continuing until roughly a week after weaning of their piglets. Livers of gilts and their piglets were assessed for different histopathological changes, apoptosis, and proliferation activity of hepatocytes. On histopathology, gilts fed the experimental diet had a statistically significant increase in hepatocellular necrosis and apoptosis (*p* = 0.0318) as well as sinusoidal leukocytosis with inflammatory infiltrates of hepatic lobules (*p* = 0.0004). The amount of interlobular connective tissue in the liver of experimental gilts was also significantly decreased (*p* = 0.0232), implying a disruption in the formation of fibrous connective tissue. Apoptosis of hepatocytes and of cells in hepatic sinusoids, further assessed by the terminal deoxynucleotidyl transferase dUTP nick-end labelling (TUNEL) assay, showed a statistically significant increase (*p* = 0.0224 and *p* = 0.0007, respectively). No differences were observed in piglet livers. These results indicated that *Fusarium* mycotoxins elicited increased apoptosis, necrosis, and inflammation in the liver of gilts, but caused no effects on the liver of piglets at these concentrations.

## 1. Introduction

Mycotoxins are toxic secondary metabolites produced by many filamentous fungi. The most important fungi causing frequent and problematic contamination of human food and animal feed belong to the fungal genera of *Fusarium*, *Aspergillus*, and *Penicillium*. Maize is considered the most susceptible crop for mycotoxin contamination and rice the least susceptible one [1].

*Fusarium* fungi produce a variety of toxic secondary metabolites, which are not essential to fungal growth but can induce several adverse effects in livestock [2]. The most toxicologically important *Fusarium* toxins are fumonisins (FBs), zearalenone (ZEN), and trichothecenes, such as deoxynivalenol (DON), nivalenol (NIV), diacetoxyscirpenol (DAS), and T-2 toxin [3]. Both in vitro and in vivo studies have demonstrated that toxicokinetics, bioavailability, and the mechanisms of action of these substances vary depending on the species involved [4]. ZEN causes reproductive abnormalities in pigs and ruminants and DON is well known for being a potent feed intake inhibitor in pigs [5,6]. Next to these well-known *Fusarium* mycotoxins, there are also several unregulated, so-called emerging mycotoxins, which frequently occur in agricultural products. One of them is fusaric acid (FA), which is found in several types of cereal grain and mixed feeds. This mycotoxin needs to be further investigated in vitro and in vivo because its neurochemical effects and possible synergistic effects with other mycotoxins, especially DON and FBs, may pose a problem to humans and livestock [7].

Besides aflatoxins (AFs) and ochratoxins, which are not *Fusarium* mycotoxins, FBs, ZEN, and trichothecenes, especially DON, are considered highly important in food safety and public health due to their widespread occurrence and toxicity. In people, chronic exposure to mycotoxins, even at low levels, may lead to adverse effects in different organs, such as the liver, kidneys, and immune system [8,9].

Due to the frequent presence of several different mycotoxins in grain and animal feed, widespread reports of co-contamination are of great potential significance [10]. A global survey indicated that 72% of samples of feed and feed raw materials are positive for at least one mycotoxin and 38% are co-contaminated [11], whereas several studies in European countries, simultaneously analyzing 20 or more mycotoxins, have shown a remarkable 44–100% of such samples to be co-contaminated with more than one mycotoxin [12]. A recent study that included 524 worldwide finished pig feed samples detected more than 235 different metabolites, including regulated mycotoxins, emerging mycotoxins, and modified/masked mycotoxins. DON was detected in 88% of the samples, mostly from the Northern Hemisphere, with a median concentration of 0.206 mg/kg of feed. All DON-contaminated samples were co-contaminated by other mycotoxins, the second most common being ZEN with a median concentration of 0.018 mg/kg of feed, while FA was not among the 60 most prevalent fungal metabolites [13].

Concomitantly occurring mycotoxins can have antagonistic, additive, or synergistic effects [14], but very little is known about their potential interactive toxic effects [2]. Even though the results from the global survey indicated that the *Fusarium* mycotoxins DON, FBs, and ZEN contaminated 55%, 54%, and 36% of feed and feed ingredients, respectively, most samples complied with even the most rigorous European Union regulations or recommendations on the maximal tolerable concentrations of individual mycotoxins [11]. Currently, the European Commission’s recommendation and its amendment on the presence of DON, ZEN, ochratoxin A, T-2 and HT-2, and FBs in products intended for animal feeding suggest that compound feed for piglets and gilts does not exceed 0.9 mg of DON/kg, 0.1 mg of ZEN/kg, 0.05 mg of ochratoxin A/kg and 5 mg of fumonisins B1 + B2/kg [15,16]. It is therefore of great importance to determine the effects of co-contaminating *Fusarium* mycotoxins, especially at naturally occurring concentrations, as well as concentrations lower than the accepted tolerance concentrations for individual mycotoxins.

Pigs are especially interesting for further research because they are one of the most sensitive animal species for *Fusarium* mycotoxins, especially ZEN and trichothecenes, such as DON and T-2. They are usually fed a cereal-rich diet, which can expose them to higher levels of these mycotoxins. As they are physiologically very similar to humans, they can serve as a good translational animal model, especially due to their similarities in the intestinal tract [17]. The effects of these toxins partly depend on their absorption, distribution, metabolism, and excretion (ADME processes) by the animal species in question. As the ADME processes seem to be qualitatively quite similar between pigs and humans, pigs can be very useful in the risk assessment of mycotoxins and for establishing legal limits of mycotoxins [18].

Research on the effects of feeding pigs with *Fusarium* mycotoxin co-contaminated feed has been ongoing for over 30 years, providing insight in various aspects. These studies often emphasized the zootechnical, hematological, biochemical, toxicological, and immunological parameters [19,20,21,22,23], whereas others also investigated histological changes in various organs with or without the aid of immunohistochemistry [14,24,25,26,27,28,29,30,31,32,33,34,35] or even examined gene expression profiles [36,37,38].

Since *Fusarium* mycotoxins are such a common contaminant and clearly have effects on different animal species, it is also of interest whether they have detrimental effects on the offspring. Some studies have analyzed the transfer of single or multiple *Fusarium* mycotoxins from sows to their offspring, implying that these can cause indirect effects via a decreased feed intake and via direct effects of diaplacentar transfer of ingested mycotoxins to the developing fetuses [20,21,29,30,31,39,40].

The liver is an important site of *Fusarium* mycotoxin metabolism [18]. DON’s effects on liver have been investigated by studies evaluating biochemical, functional, histopathological parameters [33,34,41,42,43] and even gene expression profiles [38].

The aim of this study was therefore to determine whether feed containing naturally occurring concentrations of DON, ZEN, and FA would elicit histopathological changes, a difference in the number of apoptotic cells, and the proliferation index in the liver of gilts and their suckling piglets.

## 2. Materials and Methods

### 2.1. Research Design

This study was conducted on samples retrieved from the experiment approved by the Veterinary Administration of the Republic of Slovenia and described in detail by Jakovac-Strajn et al. [22]. In summary, the experiment included 10 gilts that were fed an experimental diet containing maize naturally contaminated with *Fusarium* mycotoxins, 10 gilts that were fed a control diet, and the offspring of both groups. The gilts were daily fed 3.5 kg of the diet during gestation and 6 kg of the same diet from the day of farrowing until weaning. The gilts from the experimental group consumed significantly less than the control group, but the average bodyweight was not significantly different even at the end of the experiment. At the start of the experiment, the gilts were at 89 ± 2 days of gestation, and they remained in the experiment for a total of 54 ± 1 day. The farrowing in both groups started 24 to 27 days after the start of the experiment and the piglets were weaned at 21 days of age. No antimicrobials were given to either the gilts or their piglets during the experiment.

The experimental diet contained 5.08 mg DON, 0.09 mg ZEN, and 21.6 mg FA per kg of feed. The control diet contained 0.29 mg DON per kg of feed, whereas ZEN (<0.02 mg/kg) and FA (<0.77 mg/kg) were below their detection limits. The concentrations of aflatoxin B1 (<0.2 µg/kg), 15-ADON, NIV, fusarenon-X, DAS, T-2 toxin, HT-2 toxin (<0.05 µg/kg), ochratoxin A, and fumonisins B1, B2, and B3 (<10 µg/kg) were also measured in both diets, but they were all below their detection limits, these being indicated in parentheses.

In order to collect organs for further examination, a single 7-day-old suckling piglet was randomly selected from each of the 20 litters and killed by lethal injection of T-61 solution (embutramide/mebezonium iodide/tetracaine hydrochloride, Intervet, Unterschleißheim, Germany), whereas all the gilts were killed by captive bolt and exsanguination 5 to 8 days after weaning of the remaining piglets in the litters. Afterwards, liver samples were immediately collected, fixed in 10% phosphate buffered formalin and routinely embedded in paraffin blocks. Liver samples from two killed suckling piglets, one from each group, were inappropriate for further processing.

### 2.2. Histopathology of the Liver of Gilts and Their Suckling Piglets

Histopathological examination of 4 μm thick tissue sections of formalin-fixed paraffin-embedded (FFPE) liver samples stained with hematoxylin and eosin (H&E) was conducted using light microscopy. Several different histopathological changes were assessed in the liver: irregularity of hepatic cords, fibrosis, sinusoidal leukocytosis with inflammatory infiltrates of hepatic lobules, portal tract inflammatory infiltrates, hepatocytes with vacuolar or granular cytoplasm, hepatocellular necrosis and apoptosis, markedly enlarged hepatocytes (hepatocellular megalocytosis), markedly enlarged hepatocellular nuclei (hepatocellular megakaryosis), biliary hyperplasia, dilatation and thickening of blood vessels, and thrombosis of blood or lymphatic vessels.

Each assessed histopathological change was graded for its intensity and extent. The intensity of the histopathological changes was assigned one of the following scores: 0—not present, 1—mild, 2—moderate, and 3—severe. The extent of the histopathological changes was assigned one of the following scores: 0—not present, 1—minimal (0 to <5% of the tissue section), 2—mild (5 to <15% of the tissue section), 3—moderate (15 to <40% of the tissue section) and 4—severe (40% or more of the tissue section). The assigned intensity and extent score were then multiplied to obtain the final score for each histopathological change in the tissue section of each liver sample from both the gilts and their piglets.

### 2.3. Detection of Apoptotic Cells in the Liver of Gilts and Their Suckling Piglets

For the detection of apoptotic cells in 4 μm thick FFPE tissue sections of liver from both the gilts and their piglets, we performed the terminal deoxynucleotidyl transferase dUTP nick-end labelling (TUNEL) assay using a commercial kit (ApopTag^®^ peroxidase in situ apoptosis detection kit; Chemicon, Temecula, CA, USA) according to the manufacturer’s instructions. Finally, the tissue sections were counterstained with Mayer’s hematoxylin and coverslipped. Tissue sections of porcine kidney incubated with RQ1 RNase-Free DNase (M6101; Promega, Madison, WI, USA) were used as the positive control, and tissue sections of porcine kidney that were only incubated with the label solution (without terminal deoxynucleotidyl transferase) served as the negative control.

Using light microscopy, we counted TUNEL-positive cells in 30 randomly selected high-power fields (HPF), and also noted whether they were apoptotic hepatocytes or apoptotic cells in hepatic sinusoids. For hepatocytes to be considered TUNEL-positive, they had to have a clearly stained nucleus. Similarly, apoptotic cells in hepatic sinusoids had to exhibit moderate to marked nuclear staining.

### 2.4. Determining the Proliferation Index in the Liver of Gilts and Their Suckling Piglets

The proliferation activity of hepatocytes was evaluated on 4 μm thick FFPE tissue sections of liver from both the gilts and their piglets using immunohistochemical labelling with the mouse monoclonal antibody raised against human Ki-67 antigen, clone MIB-1 (Dako, Glostrup, Denmark), which was diluted 1:75. The antigen retrieval was performed by microwave treatment at a medium power (550 W) for 15 min in ethylenediamine-tetraacetic acid (EDTA) with a pH of 8.0. The tissue sections were then incubated with primary antibodies for 1 hour at room temperature in a humid chamber. Endogenous peroxidase activity was quenched in the peroxidase-blocking solution Dako REALTM (Dako, Glostrup, Denmark) for 30 min at room temperature. The visualization kit Dako REALTM EnVision^TM^ Detection System Peroxidase/DAB+, Rabbit/Mouse (Dako, Glostrup, Denmark) was applied according to the manufacturer’s instructions. Finally, the tissue sections were counterstained with Mayer’s hematoxylin and coverslipped. Tissue sections of porcine spleen were used as the positive control, and tissue sections of porcine liver that were not treated with primary antibodies served as the negative control. From one of the experimental gilts, a tissue section of the liver was not acquired due to lack of adequate FFPE tissue.

The proliferation index of hepatocytes was calculated as the rate of Ki-67-positive nuclei in a total of 1000 counted nuclei in the tissue sections of liver under a light microscope.

### 2.5. Morphometrical Evaluation of Interlobular Connective Tissue in the Liver of Gilts

The amount of interlobular connective tissue was measured in 4 μm thick FFPE tissue sections of liver samples only from the gilts. The tissue sections were stained with Goldner’s Masson trichrome stain to clearly depict fibrous connective tissues under a light microscope coupled with a digital camera. Using the software program NIS-Elements Basic Research (Nikon Instruments Inc., Tokyo, Japan), five consecutive microphotographs at HPF were made for each tissue section and represented the area of measurement. The microphotographs were then converted into a binary-colored output by marking pixels that belonged to either interlobular connective tissue or parenchyma, thus enabling automated detection of interlobular connective tissue. The amount of interlobular connective tissue was expressed as the area fraction of the corresponding pixels out of the total number of pixels in the area of measurement. When necessary, the automatically detected areas of interlobular connective tissue were corrected manually.

### 2.6. Statistical Analysis

For statistical analysis, we used the R statistical software, version 3.6.2 (R Foundation for Statistical Computing, Vienna, Austria) [44]. The obtained results for the experimental and control groups of both the gilts and their piglets are presented with basic descriptive statistics. The Shapiro–Wilk test was used to assess the normality of the variables. For both gilts and piglets, the differences between the experimental and control group were analyzed with the two-tailed Mann–Whitney U test because most of the variables had a non-normal distribution. The correlations between histopathological changes, the number of apoptotic cells and the proliferation index were assessed separately for the gilts and their piglets with Spearman’s rank correlation coefficients and Holm’s adjusted *p*-values. Statistical significance was determined as *p* < 0.05, and 0.05 ≤ *p* < 0.1 was marginally significant.

## 3. Results

### 3.1. Histopathology of the Liver of Gilts and Their Suckling Piglets

**Figure 1:**
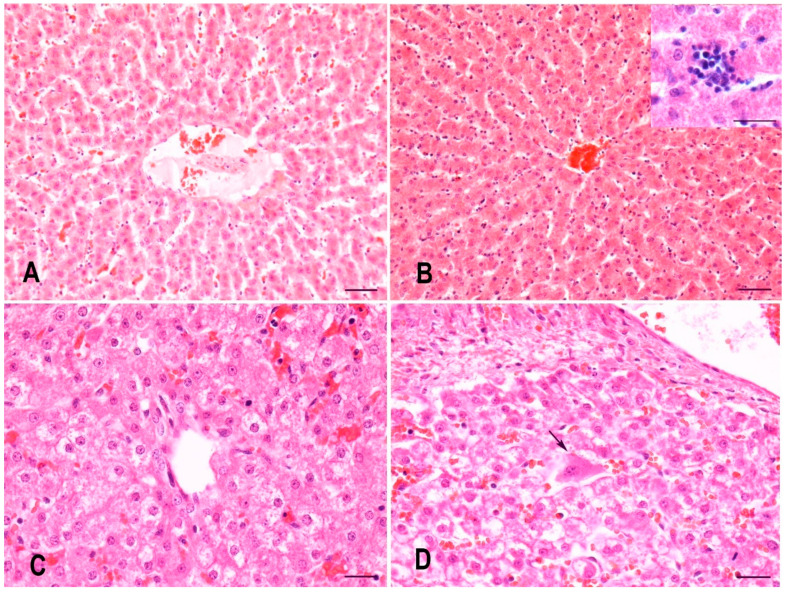
Representative microphotographs of the liver of gilts and their piglets: (**A**) gilt from the control group, (**B**) gilt from the experimental group with increased sinusoidal leukocytosis with inflammatory infiltrates of hepatic lobules (inset depicting the latter), (**C**) piglet from the control group with hepatocytes diffusely exhibiting vacuolar or granular cytoplasm, (**D**) piglet from the experimental group with hepatocytes diffusely exhibiting vacuolar or granular cytoplasm and a single hepatocyte exhibiting megalocytosis and megakaryosis (arrow). H&E, bar = 50 µm.

In gilts, seven assessed histopathological changes were observed on H&E-stained liver sections: fibrosis, sinusoidal leukocytosis with inflammatory infiltrates of hepatic lobules, portal tract inflammatory infiltrates, hepatocytes with vacuolar or granular cytoplasm, hepatocellular necrosis and apoptosis, hepatocellular megalocytosis, and hepatocellular megakaryosis. Overt fibrosis was only observed in the liver of one gilt from the control group, but even this was mild based on its final score of 1 and was not statistically significant in comparison with the experimental group (*p* = 0.3681). The remaining six histopathological changes also had low final scores with a maximum score of 2, and in one experimental gilt, hepatocytes with vacuolar or granular cytoplasm reached a score of 4 (Table 1). Final scores for hepatocellular necrosis and apoptosis (*p* = 0.0318) and sinusoidal leukocytosis with inflammatory infiltrates of hepatic lobules (*p* = 0.0004) were significantly higher in the experimental group compared with the control group (Figure 1A,B), whereas portal tract inflammatory infiltrates (*p* = 0.4539) showed no statistically significant differences. Additionally, hepatocellular necrosis and apoptosis and sinusoidal leukocytosis with inflammatory infiltrates of hepatic lobules were strongly correlated (Spearman’s ρ = 0.73, *p* = 0.0226). The inflammatory infiltrates were composed of different proportions of lymphocytes, plasma cells, neutrophils, eosinophils, and, occasionally, macrophages. Hepatocytes with vacuolar or granular cytoplasm (*p* = 0.3681), hepatocellular megalocytosis (*p* = 1.0000), and hepatocellular megakaryosis (*p* = 1.0000) did not show statistically significant differences between both groups.

In piglets, only three assessed histopathological changes were observed on H&E-stained liver sections, these being hepatocytes with vacuolar or granular cytoplasm, hepatocellular megalocytosis, and hepatocellular megakaryosis. Hepatocellular megalocytosis and hepatocellular megakaryosis had low final scores that did not exceed a final score of 1, whereas hepatocytes with vacuolar or granular cytoplasm had a final score ranging between 0 and 12 (Table 2). Similar to what was observed in gilts, hepatocytes with vacuolar or granular cytoplasm (*p* = 0.1981), hepatocellular megalocytosis (*p* = 1.0000), and hepatocellular megakaryosis (*p* = 1.0000) showed no statistically significant differences between both groups (Figure 1C,D).

Several assessed histopathological changes, namely, irregularity of hepatic cords, biliary hyperplasia, dilatation and thickening of blood vessels, and thrombosis of blood or lymphatic vessels, did not occur in any of the liver samples from either the gilts or their piglets.

### 3.2. Apoptosis and Proliferation Index in the Liver of Gilts and Their Suckling Piglets

**Figure 2:**
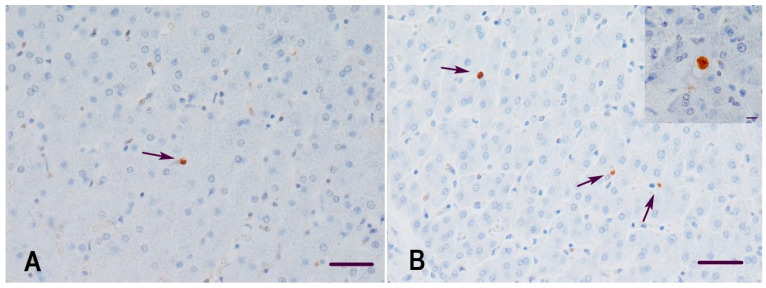
Representative microphotographs of apoptosis in the liver of gilts: (**A**) gilt from the control group with a single apoptotic cell in a hepatic sinusoid (arrow), (**B**) gilt from the experimental group with increased numbers of apoptotic cells in hepatic sinusoids (arrows) and an apoptotic hepatocyte (inset). TUNEL, counterstained with Mayer’s hematoxylin, bar = 50 µm.

The cumulative number of apoptotic hepatocytes was significantly higher in the experimental group of gilts compared with the control group (*p* = 0.0224) and an even more significant difference was observed for the apoptotic cells in hepatic sinusoids (*p* = 0.0007) (Figure 2A,B). Moreover, the apoptotic cells in hepatic sinusoids were marginally significantly but strongly correlated with sinusoidal leukocytosis with inflammatory infiltrates of hepatic lobules (Spearman’s ρ = 0.69, *p* = 0.0535). The apoptotic cells in hepatic sinusoids were most likely lymphocytes, based on their morphology, but double immunohistochemical labelling was not attempted to further clarify this. There was no statistically significant difference in the proliferation index of hepatocytes between the two groups of gilts (*p* = 0.6901) (Table 3).

In piglets, there were no statistically significant differences in the cumulative number of apoptotic hepatocytes (*p* = 0.1265) and apoptotic cells in hepatic sinusoids (*p* = 0.8581) between the two groups. No statistically significant difference in the proliferation index of hepatocytes was seen when comparing both groups of piglets (*p* = 0.1069) (Table 4). Nevertheless, the proliferation index of hepatocytes was found to be strongly correlated with hepatocytes with vacuolar or granular cytoplasm (Spearman’s ρ = 0.74, *p* = 0.006).

### 3.3. Interlobular Connective Tissue in the Liver of Gilts

**Figure 3:**
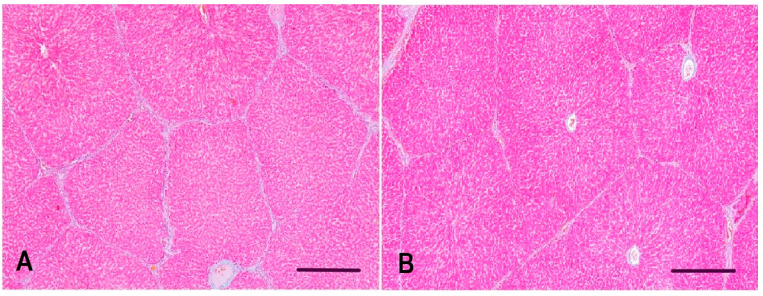
Representative microphotographs of hepatic lobules and surrounding interlobular connective tissue of gilts: (**A**) gilt from the control group with an expected amount of interlobular connective tissue and (**B**) gilt from the experimental group with a decreased amount of interlobular connective tissue in comparison with the control group. Goldner’s Masson trichrome stain, bar = 100 µm.

**Figure 4:**
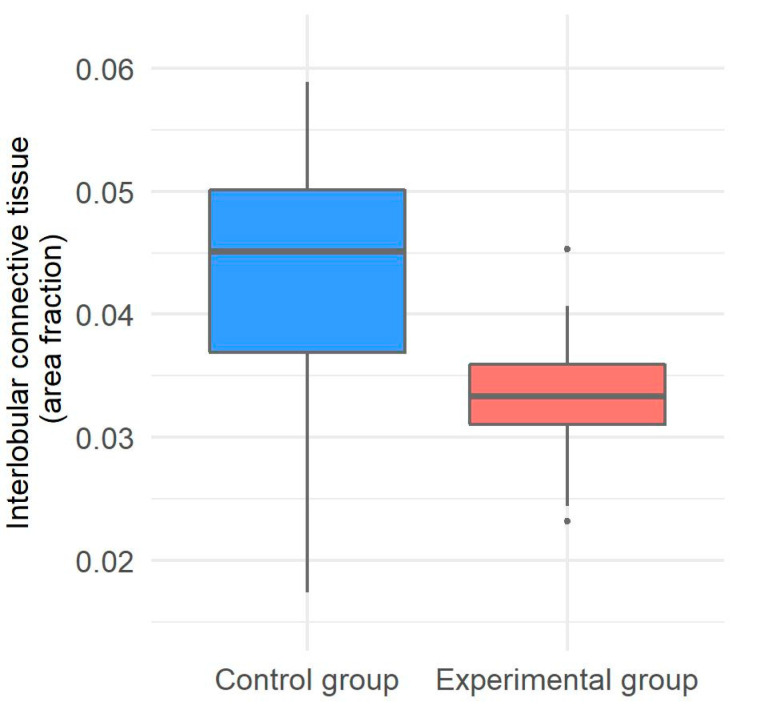
Distribution of interlobular connective tissue in the liver of the control and experimental groups of gilts with a statistically significant difference.

Morphometrical analysis of interlobular connective tissue was only implemented on liver samples from both groups of gilts. Subjectively, the interlobular connective tissue in the control group of gilts appeared to be of the expected thickness, whereas in the experimental group, it appeared mildly decreased; therefore, automated detection was important to decrease subjective bias. The interlobular connective tissue in the experimental group appeared to have decreased amounts of collagen fibers, therefore forming narrower bands of fibrous connective tissue among hepatic lobules (Figure 3). The amount of interlobular connective tissue proved to be significantly lower in the experimental group compared with the control group of gilts (*p* = 0.0232) (Figure 4).

## 4. Discussion

Mycotoxin co-contamination of finished pig feed is more common than single mycotoxin contamination [13], emphasizing the importance of investigating the effects of such naturally contaminated feeds, even when mycotoxins are detected below the accepted tolerated concentrations for individual mycotoxins. A review from Escrivá et al. [17] showed that in vivo toxicity studies of *Fusarium* mycotoxins had become much more frequent in the decade between 2003 and 2014, thus highlighting their importance. These mycotoxicoses can manifest as acute diseases with high morbidity and death or as chronic diseases, reduced animal productivity, and decreased resistance to pathogens [45].

In the present study, gilts were fed a diet containing maize naturally contaminated with *Fusarium* mycotoxins from roughly the beginning of the last quarter of their pregnancy until a week after the weaning of their piglets (a total of 54 ± 1 day). Our experimental diet contained three *Fusarium* mycotoxins, namely DON (5.08 mg per kg of feed), ZEN (0.09 mg per kg of feed), and FA (21.6 mg per kg of feed). Undertaking this approach, we assumed that the presenting liver pathology of both gilts and their piglets would mimic chronic exposure to mycotoxins in a typical pig production setting.

A detailed investigation into liver histopathology revealed a statistically significant difference in hepatocellular necrosis and apoptosis as well as sinusoidal leukocytosis with inflammatory infiltrates of hepatic lobules. Additionally, these two histopathological changes were strongly correlated, and they likely represented recruitment of inflammatory cells to sites of hepatocellular necrosis. Since the inflammatory cells were composed of a mixed population of neutrophils, eosinophils, lymphocytes, and, occasionally, macrophages, the observed rise in sinusoidal leukocytes could also be an indicator of a concurrent inflammatory process, knowing that DON is suspected to raise the susceptibility to infection and chronic diseases [46].

In humans and animals, the toxic effects of DON include emesis and anorexia, alteration of intestinal and immune functions, reduced absorption of nutrients, and elevated susceptibility to infection and chronic diseases [46]. As DON can induce such a variety of toxic effects, it is difficult to interpret whether liver pathology observed in the in vivo experiments was mostly due to direct hepatotoxic effects or significantly aggravated by indirect effects related to reduced nutrient absorption and overall decreased food intake.

Some studies have shown no changes in liver morphology when DON was either the sole potential toxic factor or in combination with another mycotoxin [30,31,40,42,43], but that was not the case in all studies. When piglets received diets either mono-contaminated with DON (1.5 mg per kg of feed) or multi-contaminated with DON (2 or 3 mg per kg of feed), ZEN (1.5 mg per kg of feed), and NIV (1.3 mg per kg of feed), the most prominent histopathological features were disorganization of hepatic cords, cytoplasmic vacuolization of hepatocytes, and megalocytosis. Piglets fed the co-contaminated diet with the higher dose of DON also exhibited focal necrosis in the liver [33]. In previous studies, feed co-contaminated with DON and ZEN often elicited microscopic changes in the liver. Chen et al. [28] fed pigs a diet containing 1 mg of DON and 0.250 mg of ZEN per kg of feed and mentioned blood vessel thickening and dilation as the only histopathological finding in the liver but did not provide a clear grading scheme or statistical analysis. When feeding prepuberal gilts for 35 days with wheat containing increasing concentrations of DON and ZEN, the amount of intracytoplasmic glycogen decreased in a dose-dependent manner, whereas hemosiderin deposition increased. Wheat containing the highest doses of DON (6.1 mg or 9.57 mg per kg of feed) and ZEN (0.235 mg or 0.358 mg per kg of feed) also elicited a statistically significant increase in the thickness of interlobular connective tissue [27]. On the other hand, a similar study, where pregnant sows were exposed to a concentration of 4.42 mg DON and 0.048 mg ZEN per kg of feed for 35 days, did not show any difference in thickness of interlobular connective tissue [29], whereas a slight difference was observed in pregnant sows receiving a concentration of 9.57 mg DON and 0.358 mg ZEN per kg of feed for 35 days [30]. Another study assessed the effects of feeding gilts for 1, 3, or 6 weeks with either DON at a dose of 0.012 mg/kg body weight (BW) per day, ZEN at 0.04 mg/kg BW, or a mixture of DON and ZEN. Histologically, several changes were observed in the liver, especially in gilts receiving DON and ZEN, such as increased thickness of perilobular connective tissue, increased total microscopic liver score, dilation of hepatic sinusoids, temporary changes in glycogen content, and increased iron accumulation in hepatocytes [34].

Interestingly, a statistically significant decrease in interlobular connective tissue was observed in our experimental group of gilts when automated detection was used on slides stained with Goldner’s Masson trichrome stain. Our finding is in contrast with previous studies because in those cases, experimental animals had an increased amount of interlobular connective tissue in the liver [27,30,34]. A recent study on collagen and elastin content in skin of mink receiving DON-contaminated feed showed a decrease in type III (immature) collagen when mink received DON at a concentration of 1.1 mg/kg of feed and complete absence of type III (immature) collagen when mink received a dose of 3.7 mg/kg DON in feed with or without 0.05% bentonite [47]. As interlobular connective tissue in pig liver contains both type I and type III collagen [48], the observed decrease in interlobular connective tissue in our study may have been due to DON influencing the expression of fibrous collagens. Further investigation would be needed to confirm or refute this assumption.

Cell culture experiments on porcine hepatocytes have shown that DON causes morphological and functional disorders in hepatocytes. Cell death of hepatocytes occurred in a dose-dependent manner and exhibited morphological changes characteristic of apoptosis. Apoptosis was further confirmed by consistently TUNEL-positive nuclei and increased activity of caspase-3, a key enzyme in apoptotic cell death [49]. Our study showed a significantly increased number of apoptotic cells in the liver of experimental gilts; an increase was observed for hepatocytes and even more so for cells in hepatic sinusoids. The apoptotic cells in hepatic sinusoids were most likely lymphocytes, based on their morphology, but we did not attempt double immunolabelling to confirm this. Similarly, piglets intravenously injected with DON at a concentration of 1 mg/kg body weight displayed systemic apoptosis of lymphocytes in lymphoid tissues as well as hepatocytes, thereby proposing a hepatotoxic potential of DON next to its already known immunotoxic effect [41]. Based on these findings, we suspect that both oral and intravenous administration of DON can cause apoptosis of hepatocytes as well as circulating leukocytes.

As DON has also been associated with antiproliferative activity [50], we assessed the proliferation activity by determining the number of Ki-67-positive hepatocytes, but found no significant differences, neither in the liver of gilts nor their piglets. The same was observed in the liver of porcine fetuses when pregnant sows were exposed to DON and ZEN for 35 days during pregnancy [40], whereas an increased proliferation index was observed when piglets received feed mono-contaminated with FBs (6 mg per kg of feed) or DON (3 mg per kg of feed) and especially when co-contaminated with both FBs and DON [14].

*Fusarium* mycotoxins clearly have direct and indirect effects on the liver of pigs that are fed contaminated diets, thereby some studies have examined possible placentar transfer from sows to their offspring [20,21,29,30,31,39,40]. Some did not identify any changes in the fetuses [40] or piglets [39]. Dänicke et al. [20] suggested that the developing fetus is exposed to DON, ZEN, and their metabolites when sows are fed contaminated feed, but did not observe any histopathological changes, and Goyarts et al. [21] also found that DON and de-epoxy-DON pass the placental barrier to a significant extent. Fetuses that were exposed to DON and ZEN between the 35th and 70th day of gestation exhibited increased glycogen content and changes in the architecture of hepatocellular mitochondria, likely caused by diaplacentar toxin transfer of ingested toxins from the mother to the developing fetuses. Feed consumption did not play a role in that experiment because both the control and experimental groups received the same amount of feed per day [29]. When sows were fed a diet contaminated with DON (9.57 mg per kg of feed) and ZEN (0.358 mg per kg of feed) between the 75th and 110th day of gestation, there were no histological changes in the livers of their piglets [30]. When sows received DON and ZEN co-contaminated diet between the 63rd and 70th day of gestation, DON was detected in fetus plasma and there was a change in the proportion of their white blood cells [31]. In our study, there were no significant differences in the assessed histopathological changes, apoptosis, and proliferation index in the livers of piglets. This suggests that a decreased feed consumption by the gilts leading to a lower energy and nutrient intake or a direct effect of diaplacentar transfer of ingested mycotoxins did not cause morphologically apparent changes in the developing fetuses, possibly due to the relatively low concentrations of *Fusarium* mycotoxins in the diet fed to the pregnant gilts.

## 5. Conclusions

The present study showed that gilts fed a diet contaminated with DON, ZEN, and FA showed significantly increased hepatocellular necrosis and apoptosis as well as sinusoidal leukocytosis with mixed inflammatory infiltrates of hepatic lobules. The number of apoptotic hepatocytes and apoptotic cells in hepatic sinusoids was also significantly higher in the experimental gilts compared with the control gilts. No significant differences were observed in the livers of their piglets, suggesting that the herein utilized concentrations of *Fusarium* mycotoxins do not have detrimental effects on the liver of offspring.

## Figures and Tables

**Table 1:** The final scores for each histopathological change in the liver of control and experimental gilts presented with basic descriptive statistics.

Histopathological Change	Group	N	Min	Q_1_	Median	Q_3_	Max
Fibrosis	control	10	0	0	0	0	1
	experimental	10	0	0	0	0	0
Sinusoidal leukocytosis with inflammatory infiltrates of hepatic lobules *	control	10	0	0	0	0	0
	experimental	10	0	1	1	1	1
Portal tract inflammatory infiltrates	control	10	0	1	1	1	2
	experimental	10	0	1	1	1	2
Hepatocytes with vacuolaror or granular cytoplasm	control	10	0	0	0	0	0
	experimental	10	0	0	0	0	4
Hepatocellular necrosis and apoptosis *	control	10	0	0	0	0.75	1
	experimental	10	0	1	1	1	1
Hepatocellular megalocytosis	control	10	0	0	0	0	1
	experimental	10	0	0	0	0	1
Hepatocellular megakaryosis	control	10	0	0	0	0	1
	experimental	10	0	0	0	0	1

* Statistically significant difference between the control and experimental groups (*p* < 0.05). N—number of animals, Min—minimum, Q_1_—lower quartile, Q_3_—upper quartile, Max—maximum.

**Table 2:** The final scores for each histopathological change in the liver of control and experimental piglets presented with basic descriptive statistics.

Histopathological Change	Group	N	Min	Q_1_	Median	Q_3_	Max
Hepatocytes with vacuolar or granular cytoplasm	control	9	0	4	8	8	12
	experimental	9	0	0	4	4	8
Hepatocellular megakaryosis	control	9	0	0	0	0	1
	experimental	9	0	0	0	0	1
Hepatocellular megakaryosis	control	9	0	0	0	1	1
	experimental	9	0	0	0	1	1

N—number of animals, Min—minimum, Q_1_—lower quartile, Q_3_—upper quartile, Max—maximum.

**Table 3:** The cumulative number of apoptotic cells and the proliferation index of hepatocytes in the liver of control and experimental gilts presented with basic descriptive statistics.

	Group	N	Min	Q_1_	Median	Q_3_	Max
Apoptotic hepatocytes *	control	10	0	0	0	1	2
	experimental	9	0	1	1	2	5
Apoptotic cells in hepatic sinusoids *	control	10	0	2.5	5.5	7.5	13
	experimental	9	11	13	16	19	35
Proliferation index of hepatocytes	control	10	0	0	0	0.08	0.2
	experimental	10	0	0	0	0	0.2

* Statistically significant difference between the control and experimental groups (*p* < 0.05). N—number of animals, Min—minimum, Q_1_—lower quartile, Q_3_—upper quartile, Max—maximum.

**Table 4:** The cumulative number of apoptotic cells and the proliferation index of hepatocytes in the liver of control and experimental piglets presented with basic descriptive statistics.

	Group	N	Min	Q_1_	Median	Q_3_	Max
Apoptotic hepatocytes	control	9	0	0	1	1	2
	experimental	9	0	0	0	0	1
Apoptotic cells in hepatic sinusoids	control	9	1	5	5	8	22
	experimental	9	1	4	6	6	20
Proliferation index of hepatocytes	control	9	0.2	0.3	0.3	0.6	1.2
	experimental	9	0	0.1	0.1	0.3	5.3

N—number of animals, Min—minimum, Q_1_—lower quartile, Q_3_—upper quartile, Max—maximum.

## Data Availability

The data presented in this study are available on request from the corresponding author.
